# Identifying Influential Nodes in Complex Networks Based on Information Entropy and Relationship Strength

**DOI:** 10.3390/e25050754

**Published:** 2023-05-05

**Authors:** Ying Xi, Xiaohui Cui

**Affiliations:** Key Laboratory of Aerospace Information Security and Trusted Computing, Ministry of Education, School of Cyber Science and Engineering, Wuhan University, Wuhan 430072, China

**Keywords:** complex networks, information entropy, influential node, relationship strength, SIR

## Abstract

Identifying influential nodes is a key research topic in complex networks, and there have been many studies based on complex networks to explore the influence of nodes. Graph neural networks (GNNs) have emerged as a prominent deep learning architecture, capable of efficiently aggregating node information and discerning node influence. However, existing graph neural networks often ignore the strength of the relationships between nodes when aggregating information about neighboring nodes. In complex networks, neighboring nodes often do not have the same influence on the target node, so the existing graph neural network methods are not effective. In addition, the diversity of complex networks also makes it difficult to adapt node features with a single attribute to different types of networks. To address the above problems, the paper constructs node input features using information entropy combined with the node degree value and the average degree of the neighbor, and proposes a simple and effective graph neural network model. The model obtains the strength of the relationships between nodes by considering the degree of neighborhood overlap, and uses this as the basis for message passing, thereby effectively aggregating information about nodes and their neighborhoods. Experiments are conducted on 12 real networks, using the SIR model to verify the effectiveness of the model with the benchmark method. The experimental results show that the model can identify the influence of nodes in complex networks more effectively.

## 1. Introduction

In the information age, human society and the natural world are filled with a large number of complex networks, such as social networks, biological networks, and transportation networks. The advancement of complex network theory has enabled a better understanding of information dissemination and the formation of real-world network structures [1,2,3]. Identifying influential nodes is one of the main research directions of complex networks, and such nodes can have an important impact on the robustness and connectivity of the network. Influential node identification has been applied to many fields, such as human physiological behavior understanding [4], analysis of the urban function [5], and price strategy analysis [6].

Much of the existing literature explores the influence of nodes in networks, and there are some classical centrality methods for identifying node influence. The centrality-based approach uses the structural and location information of the node in the network to measure the influence of the node, which usually contains the local topology information, the global topology information, and the path information between the nodes. Degree centrality [7], a simple method based on local topology information, is widely used due to its ease of operation. This local centrality method posits that the more neighboring nodes a node has, the more influential it is. In addition, other common methods include clustering coefficients [8], which consider the interconnection coefficients between nodes and their neighbors, and H-index [9], which considers the magnitude of the degree of neighboring nodes. The ESCRM method [10] defines the similarity coefficients between nodes based on their interaction relationships and local structural attributes, and then ranks the nodes based on the similarity between them. Yang et al. [11] identified the influence of nodes by considering node degrees, clustering coefficients, and neighboring nodes. Classical methods based on global topological information include K-shell [12] (based on node positions in the network) and eigenvector centrality [13] (which estimates node importance based on neighbor importance). Since K-shell cannot provide fine-grained node divisions, subsequent researchers have refined it, such as Liu et al. [14], who removed redundant links to obtain a new K-shell value for each node, resulting in a more accurate influence assessment. Wang et al. [15] proposed a node-sorting algorithm based on K-shell iteration factors. Some methods rely on path information between nodes, such as closeness centrality [16], which gauges node importance by node distance. However, closeness centrality needs to calculate all node pairs in the network, which has high complexity and is not applicable to large networks. The Katz centrality [17] method assigns weights to the walks in the network and considers short walks more important than long walks. Fei et al. [18] proposed a key node identification method based on the inverse-square law, which considers that the attraction between nodes is inversely proportional to the square of the distance between nodes, and describes the strength of a node as the sum of the attraction between node pairs. However, in complex networks, the distribution and characteristics of nodes in different networks are often not the same, and it is difficult to adapt to different types of complex networks based on a single attribute. Yu et al. [19] used the node propagation entropy and combined node-clustering coefficients with the influence of first- and second-order neighboring nodes for influence identification. Zhang et al. [20] selected four node features and used information entropy for weighting to obtain the final node representation. Previous experiments [19,20,21,22,23] have shown that by using information entropy combined with the centrality of nodes approach, performance is superior to that of a single feature.

The advent of deep learning has spurred increased attention to applying deep learning methods to explore node influence. Primary deep learning algorithms for node influence ranking include graph convolutional neural networks (CNNs) and graph neural networks (GNNs). Complex networks, as a kind of graph data, need to choose a graph embedding technique to represent the nodes as low-dimensional vectors. CNNs are often used to process various graph data by setting the receptive fields to obtain the features in the network. After the development of CNNs, scholars started to use CNN [24] for the task of node ranking. By defining the sampling method of node features in a complex network, a receptive field can map the features of nodes into a subgraph so that CNN can be used to achieve efficient node ranking. Although CNN can yield results based on node local features, it does not permit nodes to aggregate neighbor information. Consequently, some researchers utilize GNNs, which obtain node representations by aggregating neighbor information, such as InfGCN [25], which uses GNN and chooses four classical centralities as input to predict the propagation impact of nodes. The method of Zhang et al. [26] first obtains the feature matrix based on the contraction matrix of the node, and then predicts the impact of the node using GCN (graph convolution networks) and GNN. However, existing methods have limitations. For instance, CNN-based methods tend to use the local adjacency matrix of nodes, and global information cannot be awarded; graph neural network methods, such as GCN, update node information mainly by aggregating neighborhood information. The existing methods do not differentiate between nodes, thus making the propagation effect less satisfactory.

In order to overcome the previous problems and make the model applicable to complex networks, this paper considers two perspectives and proposes a new graph neural network model, the RSGNN (GNN based on relationship strength) model. Firstly, in order to overcome the limitation of single attribute, two basic features—the degree value of the node and the average neighbor degree of the node—are considered in the construction of the node input features, and their weights are determined using information entropy. It can be found that the introduction of information entropy can effectively improve the model effect through parameter analysis. Secondly, to overcome the problem that previous graph neural networks do not distinguish the importance of neighboring nodes when aggregating them, a new graph neural network model is proposed. The strength of the relationship between nodes is determined by considering the degree of neighborhood overlap during message transmission so that the model not only considers the strength of the relationship between a node and its first-order neighbors but also the strength of the relationship between a node and its second-order neighbors that have common neighbors, making the model more comprehensive in capturing the propagation influence of nodes. The model is compared with the classical graph neural network model to illustrate the effectiveness of the proposed model. The experimental results show that with different infection probability coefficients, the RSGNN model is more effective in identifying the influence of nodes in 12 real networks.

The rest of the paper is organized as follows. Section 2 presents the relevant knowledge. Section 3 details how to use information entropy to construct node features and the graph neural network model proposed in this paper. Section 4 overviews a large number of comparative experiments that were carried out in 12 real networks. Section 5 concludes the paper.

## 2. Preliminaries

### 2.1. Degree

In complex networks, the simplest index to measure the influence of nodes is the degree of nodes. The number of edges of a node is regarded as the degree value of a node. The more neighboring nodes are connected to a node, the more important the node is.

### 2.2. Information Entropy

In 1948, Shannon proposed the theory of information entropy. Information entropy is used to represent the uncertainty of the source of information by quantifying the information contained in the data. Information entropy is a metric used to measure the average amount of information. Information entropy has been widely used in various fields, such as data mining and statistical analysis.

Supposing that there are *n* events, the probabilities of the events occurring in order are $p\left(x_{1}\right),p\left(x_{2}\right),\ldots ,p\left(x_{n}\right)$, and the sum of the probabilities of each event is 1, the information entropy is

(1) $$H\left(x\right)=-\sum_{i=1}^{n}p_{i}\ln p_{i}$$

When calculating the information entropy values corresponding to multiple indicators, the information entropy can be used to calculate the weights of multiple indicators, which is an objective method of assigning weights.

### 2.3. Spreading Model

The benchmark simulator allows the effectiveness of each method to be verified by the actual propagation dynamics. For the seed nodes in the network, the propagation process is simulated uniformly using Monte Carlo methods to measure the propagation impact of the nodes. In complex networks, seed nodes are usually used as the source of infection, and the propagation process is simulated by the benchmark simulator to measure the propagation impact of the nodes, using the proportional size of the number of activated nodes to the total number of network nodes, called the infection ratio.

Many studies have proposed contagion models as a way to simulate the real-world propagation process, such as the linear threshold model [27], independent cascade model [28], and epidemic model [29]. In the simulation of the spread of complex networks, the epidemic model is widely used. In this paper, we use the susceptible–infected–recovered (SIR) epidemic model [30] to examine the propagation efficiency of each node in the network.

We transformed the node influence identification into a regression task. The propagation influence of each node is calculated using the SIR epidemic model, and the obtained influence is considered as the corresponding label of each node. In the SIR epidemic model, a node has three states, according to which the nodes can be classified into three categories: susceptible, infected, and recovered. In the initial state, only the seed node is in the infected state, and the rest of the nodes are in the susceptible state and may be infected. In each round of propagation, the infectors infect the neighboring nodes in the susceptible state with the infection probability, while the infectors move the recovery probability λ to the recovery state. In the experiments of this paper, for simplicity, we set λ = 1; in other words, the infected node tries to infect its neighbor nodes in proportion to the infection rate in the process of spreading, and then turns into the recovered node. When there are no more nodes in the infected state, the propagation process is terminated. At this point, the statistical node infection ratio is used to measure the spreading impact of the node. The number of recovered nodes in the network is counted, and the proportion of such nodes to the total number of nodes is calculated and considered the percentage of infection of the seed nodes. In order to obtain more objective experimental results, several independent experiments were conducted, and the number of experimental simulations was denoted as $S_{n}$. The average proportion of infections for experiments was calculated using the following formula:

(2) $$t=\frac{\overset{S_{n}}{\underset{j=1}{\sum }}N^{r}}{S_{n}*N}$$

where *N* is the total number of nodes in the network, $N_{r}$ is the number of recovered nodes, and *t* is the proportion of final nodes infected. In this paper, the number of experimental simulations $S_{n}$ is set to 500. To prevent the infection probability from being too large, information spreading from any node can infect a large number of the network nodes, and the infection probability β is considered at the network prevalence threshold [31]:

(3) $$\beta_{th}=<k>/<k^{2}>$$

<*k*> is the average degree of the network, <$k^{2}$> is the second-order average degree of the network, and β${}_{th}$ is the network epidemic threshold. In this paper, we take β${}_{th}$ to two decimal places. Finally, the proportion of infections is considered node labels, and the input features and node labels are used to form the dataset from which the model is trained.

## 3. Proposed Method

This section provides a detailed introduction to the RSGNN model, covering three aspects: the construction of node input features by information entropy, the implementation of the message passing layer in the graph neural network, and the overall model framework. Table 1 presents frequently used symbols and their meanings within this paper.

### 3.1. Constructing Node Features by Information Entropy

The influence of a node in a complex network is closely related to its neighboring nodes. Consequently, evaluating node influence requires considering information about the node itself and its neighboring nodes. In this paper, we first obtain a list of each node’s first-order neighbors and sort them by the degree of their neighbors. The input to the RSGNN model comprises the basic features of the node and its neighboring nodes, with the first position representing the node’s basic feature and the remaining positions representing the basic features of neighboring nodes. The algorithm is described in Algorithm 1.
**Algorithm 1:** Algorithm for generating input features**Input:** network**Output:** the input features of the node1. N(u)← Get a list of neighbors for each node.2. k(u),$\bar{k(u)}$← Calculate the degree of a node and the average degree of the neighbor.3. $\omega_{1},\omega_{2}$← Calculate the weights of the node degree and the average degree of the neighborusing information entropy.4. B(u),F(u)← Construct basic features and input features for each node.

Initially, acquire the first-order neighbor list of each node, and sort the nodes by their neighbors’ degree to construct a neighbor list of nodes, $N\left(u\right)=\left(u_{1},u_{2},\ldots ,u_{m}\right)$. After obtaining the neighbor list, calculate the degree values of the nodes and the average degree of the node’s neighbors:

(4) $$\bar{k(u)}=\frac{1}{\left|N(u)\right|}\sum_{v\in N(u)}k(v)$$

The RSGNN model determines the weights of node degree values and neighbor average degrees based on information entropy, and first normalizes the node degree values and neighbor average degrees as follows:

(5) $$k\;\;{\;}^{\prime }\;\;\;\left(u\right)=\frac{k(u)-\min(k)}{\max(k)-\min(k)}$$

(6) $$\bar{k\;\;{\;}^{\prime }\;\;\;\left(u\right)}=\frac{\bar{k(u)}-\min\left(\bar{k}\right)}{\max\left(\bar{k}\right)-\min\left(\bar{k}\right)}$$

where max(k) and min(k) represent the maximum and minimum values of degree values in the network, respectively, and  $\max(\bar{k})$ and $\min(\bar{k})$ represent the maximum and minimum values of the average degree of the neighbor, respectively. The sum of the degrees of all nodes in the normalized network and the sum of the average degree of the neighbor are calculated such that $K_{sum}=\underset{u\in V}{\sum }k^{\prime }\left(u\right)$, ${\bar{K}}_{sum}=\sum_{u\in V}\bar{k^{\prime }\left(u\right)}$ so as to calculate the information entropy values corresponding to the two features, which are calculated as follows:

(7) $$H_{k}\;=-\frac{1}{\ln\;n}\sum_{u\in V}\frac{k^{\prime }\left(u\right)}{K_{sum}}\ln\frac{k^{\prime }\left(u\right)}{K_{sum}}$$

(8) $$\bar{H_{k}}\;=-\frac{1}{\ln\;n}\sum_{u\in V}\frac{\bar{k^{\prime }\left(u\right)}}{{\bar{K}}_{sum}}\ln\frac{\bar{k^{\prime }\left(u\right)}}{{\bar{K}}_{sum}}$$

where $H_{k}$ represents the information entropy value of the node degree and $\bar{H_{k}}$ represents the information entropy value of the average degree of the neighbor. Further processing of $H_{k}$ and $\bar{H_{k}}$ leads to the final weights:

(9) $$\omega_{1}=\frac{1-H_{k}}{2-H_{k}-\bar{H_{k}}}$$

(10) $$\omega_{2}=\frac{1-\bar{H_{k}}}{2-H_{k}-\bar{H_{k}}}$$

Using the calculated information entropy weight values, the node degree and the average degree of the neighbor are weighted to obtain the basic features of the node:

(11) $$B\left(u\right)=\omega_{1}*k\left(u\right)+\omega_{2}*\bar{k(u)}$$

Since the influence of a node in a complex network is closely related to its neighboring nodes, the basic features of node u, B(u), are combined with the basic features of its neighboring nodes to form the input features of the node, F(u). The input feature dimension of a node is set to 24, and if the number of neighboring nodes is less than 24, it is filled with 0. If the number of neighboring nodes is greater than 24, only the basic features of the first 23 neighboring nodes are taken. For example, for node u, its input feature is F(u)=[B(u),B(u1),…,B(u23)]. It can be observed that the final input feature F(u) contains information related to the node, first-order neighbor nodes, and second-order neighbor nodes simultaneously, allowing the model to comprehensively obtain node information.

### 3.2. Message Passing Layer

The strength of the relationship between nodes needs to be considered when identifying node influence because different neighboring nodes tend to have different influences on the target node. Previous graph neural network approaches have often overlooked this aspect. In this paper, drawing on existing literature [32], we define the relationship strength between nodes as the degree of overlap between the neighbors of two nodes, using the number of common neighbors as a reference indicator to measure the relationship strength. In this paper, we argue that the higher the number of common neighbors among nodes, the more susceptible they are in message propagation. Based on this idea, this paper proposes a new graph neural network algorithm to calculate node influence.

**Figure 1:**
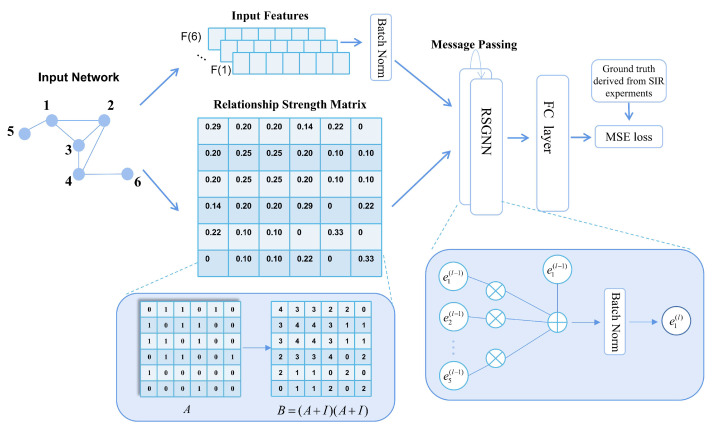
The RSGNN model is divided into three main steps: (**1**) constructing the input features of the nodes and obtaining the relational strength matrix of the network, (**2**) inputting the input features and relational strength matrix into the two message passing layers, and (**3**) obtaining the final representation of the nodes through the fully connected layer.

As shown in Figure 1, the first-order neighbors of node 1 are nodes 2, 3, and 5. As can be seen from Figure 1, nodes 2 and 3 have common neighbors with node 1, are more closely related, and have a greater influence on node 1 when information is disseminated; while node 4 is not a first-order neighbor of node 1, it has two common neighbors with it (nodes 2 and 3) and therefore also has some influence on it.

The adjacency matrix of the network is defined as *A*. If $A_{ij}$ is 1, it indicates that there are edges between node i and node j, and they are each other’s neighbor nodes. The number of common neighbors of nodes i and j is defined as $C_{ij}$. From the properties of the adjacency matrix, we know that by using $A_{i.}$ to denote the i-th row of the adjacency matrix and $A_{.j}$ to denote the j-th column of the adjacency matrix, the inner product of $A_{i.}$ and $A_{.j}$ is the number of common neighbors of node i and node j $C_{ij}$. To avoid missing first-order neighbors that have no other common neighbors, for first-order neighbor nodes, the node relationship strength is considered to be the number of common neighbor nodes $C_{ij}$ plus 2, and for second-order neighbor nodes, the node strength is the number of common neighbor nodes. The relationship strength matrix is defined as follows:

(12) R=(A+I)(A+I)

in which

(13) $$R_{ij}=\left\{\begin{array}{c} k(i)+1,\;i=j\\ C_{ij}+2,\;j\in N\left(i\right)\;\\ C_{ij},\;j\notin N\left(i\right)\; \end{array}\right.$$

It can be observed that in the relationship strength matrix *R*, information about the node itself is considered, as well as information about the node’s first-order neighbors and its second-order neighbors, where there are common neighbors. Denoting the sum of the elements of the i-th row of the relationship strength matrix *R* as $S_{i}$, the influence of neighbor node j on node i is expressed as

(14) $$m_{i\leftarrow j}=\frac{e_{j}*R_{ij}}{\sqrt{S(i)S(j)}}$$

where $e_{j}$ is the current layer feature of node j and $m_{i\leftarrow j}$ is considered the influence of node j on node i. The influence of neighboring nodes on the current node is obtained by considering the strength of the relationship between the neighboring nodes and the current node. The information of the neighboring nodes in the node domain is aggregated and combined with the node’s own features to obtain an updated representation of the node as

(15) $$e_{i}^{\left(l\right)}=W_{1}e_{i}^{\left(l-1\right)}+\sum W_{2}m_{i\leftarrow j}^{\left(l-1\right)}$$

where $W_{1},W_{2}$ are the two trainable parameter matrices. After each layer of message passing, the data are normalized using batch normalization to improve the training stability and convergence rates. For an intuitive view of the overall algorithm, a matrix form representation of the algorithm is provided:

(16) $$e^{\left(l\right)}=BatchNorm\left(Ie^{\left(l-1\right)}W_{1}^{\left(l\right)}+Le^{\left(l-1\right)}W_{2}^{\left(l\right)}\right)$$

(17) $$L=D^{-1/2}RD^{-1/2}$$

where *I* is a unit array of size n×n, *D* is a diagonal matrix, and $D_{ii}=S\left(i\right)$, the sum of the data in row i of the relational strength matrix *R*.

### 3.3. Model Framework

The training model process is shown in the Algorithm 2. The RSGNN model consists of an input layer, two message passing layers, and a fully connected layer. The implementation details are as follows:
**Algorithm 2:** Algorithm for training the RSGNN model.**Input:** Training networks:G**Output:** Trained model1. F ← Generate_Embeddings(G)2. t ← Calculate_Influence(G)3. R ← Generate_Relationship_Strength_Matrix4. RSGNN← Create_RSGNN_Model()5. $RSGNN^{T}$← Train_Model(RSGNN, F, t, R)6. return $RSGNN^{T}$

As shown in Algorithm 2, firstly, for each node in the network, the information entropy is calculated based on its degree and the average degree of the neighbor, and is then used as the weight to construct the basic and input features of the node.

Secondly, obtain the label of each node according to the SIR propagation model.

Thirdly, the input features of the node are normalized using batch normalization, and the relationship strength matrix is constructed for each network.

Fourthly, initialize the RSGNN model.

Fifthly, the normalized features of the node and the relationship strength matrix of the network are fed into the RSGNN model. The message-passing layer enables the nodes to fuse multi-hop neighborhood information. After, the aggregated node information is fed into the fully connected layer with an output dimension of 1. The output of the fully connected layer is considered the fraction of nodes, and the node labels obtained from the SIR model are chosen to minimize the loss function. The loss function is MSE (mean squared error), the gradient optimization function is NAdam, and the learning rate is set to 0.001.

Finally, return the trained model.

## 4. Experimental Evaluation

### 4.1. Kendall Correlation Coefficient

In this paper, the Kendall τ rank correlation coefficient [33] is chosen as a criterion for judging the different methods. The value of Kendall τ is a statistical value used to measure the correlation of two random variables. The Kendall correlation coefficient is mainly calculated by counting the number of concordant pairs and the number of discordant pairs in the two lists, assuming that in the first list, node m is ahead of node n. If node m is still ahead of node n in the second list, it is regarded as a concordant pair; otherwise, it is regarded as a discordant pair, and the calculation formula is as follows:

(18) $$\tau =\frac{N_{c}-N_{d}}{(n(n-1))/2}\;$$

where *N*${}_{c}$ is the number of concordant pairs and *N*${}_{d}$ is the number of discordant pairs. It can be seen that the more the number of concordant pairs, the more similar the ranking results of the nodes in the list. The value of Kendall τ ranges from −1 to 1. When τ is 1, it means that the two sorted lists are identical; when τ is −1, it means that the two sorted lists possess exactly the opposite correlation; and when τ is 0, it means that the two sorted lists are independent of each other.

### 4.2. Benchmark

(1) Degree centrality (DC) [7]

(19) $$\mathrm{DC}(u)=\frac{k(u)}{n-1}$$

The degree value of a node refers to the number of neighbor nodes, where *n* is the number of nodes in the network, *k*(*u*) represents the degree of the node, and DC(u) is the degree centrality of the node.

(2) The K-shell decomposition method (KS) [12]

In the K-shell decomposition method, the nodes with degree 1 and the connected edges are first removed, and the K-shell index of these nodes is set to 1. This process is repeated until there are no nodes with degree 1 in the network. Next, the nodes with degree 2 and the connected edges are removed, and the K-shell index of the nodes is set to 2. This process is repeated until there are no more nodes in the network.

(3) Betweenness centrality (BC) [34]

(20) $$\mathrm{BC}(u)=\sum_{m\neq n\neq u}\frac{g_{mn}^{u}}{g_{mn}}$$

The betweenness centrality of nodes refers to the proportion of the number of shortest paths passing through node u to all shortest paths in the network. g${}_{mn}$ is the number of shortest paths between node m and node n, and g${}_{mn}^{u}$ is the number of shortest paths passing through node u between node m and node n.

(4) PageRank centrality (PR) [35]

PageRank originated from network ranking and considers that the influence of a web page depends on the number and quality of other pages pointing to it. PageRank determines the importance of a node by considering the influence of neighboring nodes and requires global iterative calculations.

(5) RCNN [24]

The RCNN model is implemented based on CNN. The authors first find its L-1 neighbor nodes for each node, which constitute the node’s corresponding adjacency matrix, and then combine the node’s adjacency matrix and the degree to generate the node’s L-dimensional feature matrix. The RCNN model mainly contains two layers of convolution, two layers of pooling, and one layer of fully connected layers. In the first layer of convolution, the size of the convolution kernel is 5 × 5, the numbers of input channels and output channels are 1 and 16, respectively, the stride is 1, and the padding is 2. In the second layer of convolution, the convolution kernel size, stride and padding are kept the same as the first layer of convolution, the number of input channels is 16 and the number of output channels is 32. After each layer of convolution, the nodes are convolved by a 2 × 2 max pooling and finally by a fully connected layer of 32 × (L/4) × (L/4) to 1. The activation function is chosen as ReLU, and the loss function is the squared loss function.

(6) InfGCN [25]

The InfGCN model is mainly based on GCN implementation, which consists of an input layer, a GCN layer, three fully connected (FC) layers, and an output layer. InfGCN selected four centrality metrics of nodes degree, closeness centrality, betweenness centrality, and clustering coefficient as the characteristics of nodes. The input features go through one GCN layer, and the exponential linear units (ELU) is chosen as the activation function, while the authors add residuals and dropout in the GCN layer, and finally the node representation is obtained after three fully connected layers. Note that the first two fully connected layers are followed by a layer of activation function ELU.

(7) CGNN [26]

The CGNN model is mainly based on CNN and GNN. The CGNN model firstly obtains the information of node L neighbors and constructs its adjacency matrix A(x); secondly, according to the contraction algorithm, the condensed adjacency matrix B(x) can be obtained, and the feature matrix F(x) of the node is obtained through A(x) and B(x). After constructing the feature matrices of the nodes, the CGNN model is first passed through two CNN layers, each followed by an activation layer and a pooling layer. In the first layer of CNN, input channel is 1, output channel is 10, the convolutional kernel is 3 × 3, stride and padding is 1. In the second layer of CNN, the input channel is 10, the output channel is 20, and the convolutional kernel, stride and padding are the same as the first layer. The activation function of the CNN layer is LeakyRelu. The node features of 6 dimensions are then input into the two layers of GNN. After the two CNN layers, a fully connected layer is followed, whose input dimension is ((l + 1)${}^{2}$/4${}^{2}$) × 20 and output dimension is 6:

(21) $$e^{i+1}=\sigma (Ae^{i}W^{i}\;+\;b^{i})$$

In GNN, *e^i^* is the representation of the node at the i-th layer, A is the adjacency matrix of the network, and the activation function is the sigmoid function. There are two layers of the GNN model in CGNN, the *W*^0^ dimension is 6 × 2 and *b*^0^ dimension is two in the first layer, and the *W*^1^ dimension is 2 × 4 and the *b*^1^ dimension is four in the second layer. Finally, L2-norm and sigmoid are used to obtain node representation. The optimizer is Adam and the loss function is MSE.

### 4.3. Datasets Description

The BA model is a scale-free network model, in which the degrees of vertices follow a power-law distribution, and a very small number of nodes have a large number of connections. The LFR model can generate complex networks with multiple associations in which the degrees of nodes in each association follow a power-law distribution. In this paper, the BA synthetic network and the LFR synthetic network are chosen to train the model, and 12 real networks are selected to verify the model’s effectiveness. The networks in this paper are all maximally connected components of the original network, and all the networks are undirected graphs. Table 2 shows the details of the 12 real networks in the test set, where *n* denotes the number of nodes in the network, |E| denotes the number of edges in the network, <k> denotes the average degree value of the network, Δ denotes the maximum degree value of the network, C denotes the clustering coefficients of the network, and r represents the degree pearson correlation. The clustering coefficients C and the degree pearson correlation coefficient r are two statistical indicators of a network. The clustering coefficient C indicates the degree of aggregation of nodes in a network, and a higher clustering coefficient indicates that the nodes are more closely connected to each other. The degree Pearson correlation coefficient r measures the degree assortativity of the network. A higher coefficient indicates that nodes in a network tend to be connected to nodes that are similar to it, where r > 0 indicates that the network is assortative, and nodes with a higher degree tend to be connected to nodes with a higher degree, where r < 0 indicates that the network is disassortative.

In order to better demonstrate the applicability of the RSGNN model to complex networks in various domains, a number of well-known complex network datasets were selected, including social networks, flight networks, biological protein networks, power grid, collaboration networks, etc. The datasets are described as follows:

Lesmis [36]: A co-occurrence network of characters in the novel *Les Miserables*, where a node in the network represents a character in the book, and an edge represents the relationship between two characters.

Polbooks [37]: A political book network, where a node in the network represents a book, and an edge represents the simultaneous purchase of two related books.

Adjnoun [38]: A network of adjective and noun adjacencies commonly found in DickensâĂŹ novel, *David Copperfield*, where the nodes in the network indicate adjectives or nouns, while the edges indicate that both occur in any adjacent position.

Moreno [39]: This directed network captures 246 physicians in towns in Illinois, Peoria, Bloomington, Quincy and Galesburg, with one node representing a physician and the sides representing two physicians who know each other or are likely to have discussions.

Jazz [40]: A social network among jazz musicians, where the nodes represent musicians.

USAir [37]: A map of the U.S. airport route network, where the nodes in the network represent airports, and the edges indicate a route between two corresponding airports.

Netsience [38]: The co-authorship of scientists in network theory and experiments, where the vertices represent the researchers and the edges represent the co-authorship relationships.

Faa [41]: A route database obtained from the FAA’s National Flight Data Center in the United States, where the nodes represent airports or service centers, and the sides are determined by strings of preferred routes.

Hamster [41]: A social network extracted from the site hamsterster.com, where nodes represent users on the site and sides represent friendships or family ties between users.

Figeys [42]: Interaction networks between human proteins; an invaluable tool for understanding protein function.

Facebook [43]: A social network from Facebook, where nodes represent users and edges represent users’ friendships.

PowerGrid [44]: A network of information about the power grids of the western states of the United States.

**Figure 2:**
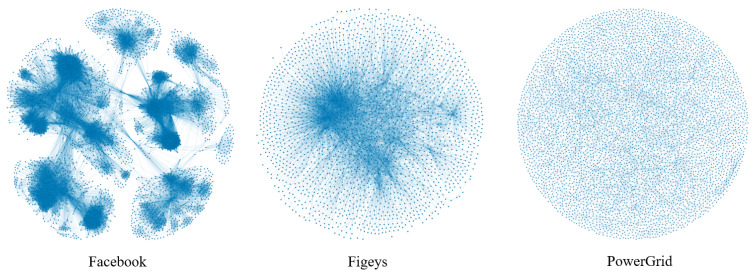
Visualization of the networks: Facebook, Figeys and PowerGrid.

**Figure 3:**
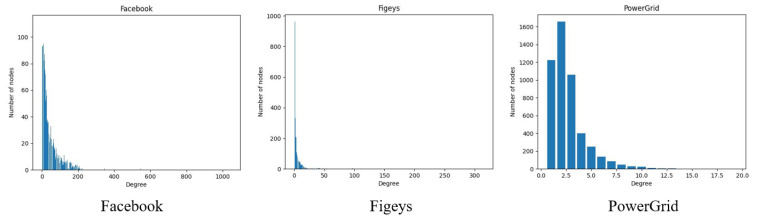
Degree distribution of the networks: Facebook, Figeys and PowerGrid.

Larger disparities between complex network datasets can be found in the real world. For example, in the social networks Facebook and Jazz, the clustering coefficients between nodes are higher than those of other types of complex networks, and in terms of correlation, both are biased toward homogeneity, which is due to the fact that in social networks, people are more closely connected to each other. In the Facebook social network, the probability that the remaining two users in a user group know each other is 51.91%, and there is a tendency for users to hug each other, whereas in the bioprotein network Figeys, the connections between nodes are sparser and the network shows heterogeneity in terms of the degree Pearson correlation coefficient. In the PowerGrid network, there are 4941 nodes, but the maximum degree value is only 19, and each node interacts with an average of 3 nodes, making the connections between nodes relatively sparse. In order to better see the differences between the networks, we show visualizations of three networks—Facebook, Figeys and PowerGrid—as shown in Figure 2. Figure 3 also shows the degree distribution of these three networks.

### 4.4. Ablation Experiment

In order to verify the effectiveness of using information entropy to combine the node degree value and the average degree of the neighbor, the node’s degree value k(u), the average degree of the neighbor $\bar{k(u)}$, and the feature B(u) constructed based on information entropy were used as the basic features of the nodes to construct model inputs. The corresponding models were named RSGNN_K, RSGNN_$\bar{K}$, and RSGNN. The Kendall correlation coefficient was used to measure the correlation between the output results of each model and the ranking results of the SIR propagation model, with higher correlation coefficient values indicating better model results. Table 3 shows the information entropy weight values in the 12 real networks, and the experimental results under the 3 models. It is worth noting that in all experimental results in this section, *p*-values are below 0.01, proving that the results are statistically significant.

In Table 3, $\omega_{1}$ is the information entropy weight corresponding to the node degree value, and $\omega_{2}$ is the information entropy weight of the first-order neighborhood average degree. In all networks, the RSGNN model based on the features obtained from information entropy fusion achieves optimal results. Additionally, by observing the weights, it can be found that the weight of the node degree value is slightly larger than the weight of the average degree of the neighbor. The larger the value of $\omega_{1}$, the more obvious the advantage of the RSGNN_K model over the RSGNN_$\bar{K}$ model; the larger the $\omega_{2}$, the more obvious the advantage of the RSGNN_$\bar{K}$ model using the average degree of the neighbor. The comparison experiments on 12 networks show that using information entropy to fuse the degrees of nodes and the average degree of the neighbor allows the model to capture more comprehensive information about the nodes, making the experimental results more valid.

### 4.5. Compared with Benchmark Methods

In this section, we compare the RSGNN model with other benchmark methods. To better evaluate the performance of the model under different infection probabilities and to observe the effect of infection rate β on the experimental results, we set the values of infection rate β/β${}_{th}$ to 1, 1.2, 1.4, 1.6, 1.8, and 2.0 for multiple experiments. We used the Kendall correlation coefficient and the SIR model to evaluate the effectiveness of each method. We compared the node ranking sequence obtained by each method with the node ranking sequence obtained by the SIR model, and the larger the correlation coefficient obtained, the closer the influence of the node is to the real influence of the node.

**Figure 4:**
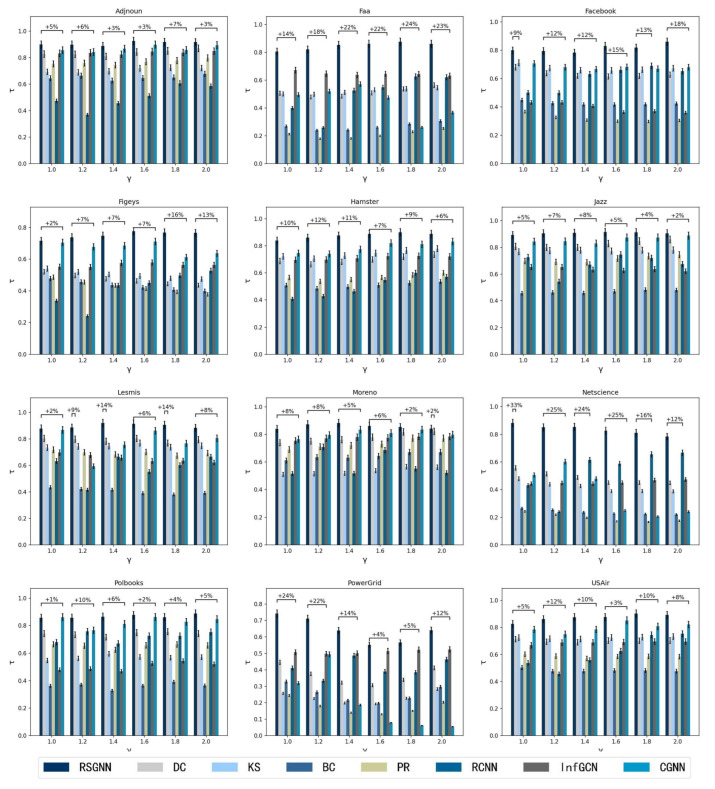
Kendall coefficient changes of RSGNN model and other models under different infection probability ratios. The scale of the horizontal axis indicates the infection probability coefficient β/β${}_{th}$, and the vertical coordinate is the Kendall correlation coefficient. In this experiment, all *p*-values are below 0.01 and, therefore, the results are statistically significant.

To verify the effectiveness of the model, 12 real networks were selected, and RSGNN was compared with other methods in this paper. Figure 4 shows the Kendall correlation coefficient values for each method in the 12 networks at different infection rate coefficients and the percentage increment of RSGNN relative to the best comparison method in each network. As shown in Figure 4, the RSGNN model outperformed in most cases, with the highest Kendall correlation coefficient as the infection rate coefficient β/β${}_{th}$ varied from 1 to 2. At the same time, the RSGNN model has a clear advantage regarding all probabilities of infection. For example, in the network PowerGrid, there are 4942 nodes, the maximum degree value of nodes is 19, most nodes have a degree distribution of 1–7, the connection between nodes is relatively sparse, and there is no hub node with a large degree value. The nodes are more uniformly distributed, and at this time, the use of degree centrality, K-shell and other methods cannot well distinguish nodes. In the neural network-based approach, the RCNN model inputs features to a degree; however, the RCNN model, using the convolutional neural network, can find the local information of nodes, so the accuracy rate is higher than the traditional method. The CGNN model inputs based on the node local adjacency matrix. In the network PowerGrid, the local adjacency matrix of nodes has less variance, and the aggregation of neighbor information shows that all nodes have the same importance, so the performance is poor. The InfGCN model has four centrality methods of nodes selected in the input features, which can mine more node features, and therefore displays higher performance than the other methods in the baseline method; however, none of these methods consider the connection strength between nodes when aggregating the neighborhood node information, so the performance is weaker than the method proposed in this paper. In the Facebook network, which has a high clustering coefficient, there are 4039 nodes with a large gap in degree distribution, with node degrees ranging from 1 to 1045. As can be seen from Figure 3, there are multiple associations in the Facebook network, with 73 nodes having degree values greater than 190, presumably because the core nodes in each community are in each association, and thus using degree centrality and the K-shell method based on the location of nodes in the network can achieve good results. In the Facebook network, from the clustering coefficient, C = 0.51 can be obtained, and the direct degree of overlap of node neighbors is larger, so this paper considers the degree of overlap of neighbors as the strength of the relationship between nodes and uses this as the basis to achieve message aggregation, which performs better than the InfGCN and CGNN methods. In the protein network Figeys, there are 2217 nodes in total, of which 962 nodes have degree 1 and 332 nodes have degree 2. Most nodes have similar degree distribution, so most methods cannot distinguish node influence well, and the neural network-based methods perform better than previous centrality methods, and the RSGNN method, which considers the strength of node relationships, outperforms the other. The experimental results demonstrate that the RSGNN model outperforms the other methods, even though it only utilizes the degree of the nodes and the average degree of the neighbor. For example, the reason for outperforming the InfGCN method, which uses the classical features of four nodes, is that the RSGNN model considers the strength of relationships between nodes to aggregate node information, which has higher stability and can identify the influence of nodes more accurately.

### 4.6. Compared with GNN Methods

In order to verify the aggregation effect of the RSGNN model, we input the features of this paper into other classical GNN models. In each graph neural network, we set up two layers of aggregation: the activation function is ReLU, and the optimizer is NAdam. We provide a brief introduction to three types of graph neural networks:

GCN (graph convolution network) [45]: The GCN model extends the convolution operation from traditional data to graph data. The node feature matrix, adjacency matrix, degree matrix, and other information of the graph network are required. The adjacency matrix of the network needs to be normalized, and the core idea is to realize the aggregation operation of node features by multiplying the adjacency matrix with the feature matrix.

GraphSAGE (graph sample and aggregate) [46]: It mainly consists of two steps, sampling and aggregation: firstly, sampling the neighbors using the connection information between nodes, and then fusing the information of the sampled neighbor nodes together through a multi-layer aggregation function.

GAT (graph attention network) [47]: When aggregating feature information, the GAT model applies the attention mechanism to determine the weight of node neighbors, and realizes the adaptive matching of the weights of different neighbor nodes so as to aggregate the neighbor nodes.

Table 4 shows the Kendall correlation coefficients between the output of the RSGNN model and the three graph neural networks and the ranking results of the SIR model in the 12 real networks. Again, in the experiments in this section, all *p*-values are below 0.01 and, therefore, the results are statistically significant.

As seen in Table 4, the RSGNN model consistently outperformed the three graph neural networks, delivering the best and most stable results. In contrast, the performance of the three graph neural network models exhibited significant fluctuations. For instance, the GCN model achieved a high result of 0.85 in the USAir network, GAT had a high result of 0.74 in the Facebook network, and GraphSAGE scored 0.82 in the Jazz network. However, in some networks, the results were below 0.5. The reasons for these disparities are as follows: The GCN model requires the adjacency matrix of the entire network structure for training and does not differentiate the strength of relationships between nodes. The GAT model relies on the weights of nodes to neighboring nodes, which can lead to overfitting. The GraphSAGE model depends on the sampling and aggregation of neighboring nodes, and the fixed number of sampled nodes may result in the loss of local node information. Furthermore, random sampling could make node features less stable. The RSGNN model, on the other hand, takes into account the strength of relationships between nodes and aggregates the information of neighboring nodes as well as the nodes’ own characteristics when disseminating information.

## 5. Conclusions

Identifying influential nodes has become a key research direction in complex networks. Existing methods tend to ignore the strength of relationships between nodes and cannot adapt to the diversity of complex networks based on a single feature. Since complex networks extracted from the real world contain less information about the weights between nodes, this paper defines the strength of relationships between nodes by considering the degree of neighborhood overlap between nodes and proposes a graph neural network model RSGNN based on the strength of node relationships to identify node influence. The model uses information entropy combined with the degree value of nodes and the average degree of the neighbor to construct the input of the model. The strength of the relationship between nodes is considered, and the information of neighboring nodes is aggregated. To verify the effectiveness of the RSGNN model, this paper uses the Kendall correlation coefficient as an evaluation metric and compares it with 7 existing methods in 12 real networks. The results demonstrate that the RSGNN model performs best under various infection probability coefficients β/β${}_{th}$. Furthermore, the input features of the RSGNN model were fed into three classical graph neural network models for comparison. The experiments revealed that the RSGNN model can effectively aggregate neighborhood information by considering the strength of the relationship of nodes and outperforms the selected graph neural network model. The RSGNN model proposed in this paper provides a new idea for identifying the influence of nodes in complex networks; however, there are still some challenges that need to be further overcome in future work. Firstly, the model proposed in this paper is only applicable to undirected graphs, and we will continue to explore its improvement in directed networks in future research; secondly, the current application scenario of the model is static complex networks, and its extension to dynamic networks can be considered in future work.

## Figures and Tables

**Table 1:** Common symbols and explanations.

Symbols	Explanation of Symbols
*V*	The set of nodes in the network
*n*	Total number of nodes
*A*	Adjacency matrix of the network
*I*	Unit matrix of n×n dimensions
k(u)	Degree value of node u
$\bar{k(u)}$	Average neighbor degree of node u
N (u)	The list of neighbors of node u
B(u)	The basic feature of node u
F(u)	The input feature of node u
$\omega_{1}$	Weighting of the degree
$\omega_{2}$	Weighting of the average degree of the neighbor
$C_{ij}$	Number of common neighbors of node i and node j
*R*	Relationship strength matrix

**Table 2:** The statistical indicators of the test data set.

Network	n	|E|	<k>	Δ	C	r
Adjnoun	112	425	8	49	0.16	−0.12
Facebook	4039	88,233	44	1045	0.52	0.06
Hamster	1788	12,476	14	272	0.09	−0.09
Jazz	198	2742	28	100	0.52	0.02
Faa	1226	2410	4	34	0.06	−0.01
Figeys	2217	6418	6	314	0.01	−0.33
Lesmis	77	255	7	36	0.5	−0.16
Moreno	117	465	8	26	0.17	−0.08
Netscience	379	914	5	34	0.43	−0.08
PowerGrid	4941	6594	3	19	0.1	0.00
Polbooks	105	442	9	25	0.35	−0.13
USAir	332	2126	13	139	0.4	−0.21

**Table 3:** The Kendall correlation coefficient of the model with different input features.

Network	$\omega_{1}$	$\omega_{2}$	RSGNN_*K*	RSGNN_$\bar{K}$	RSGNN
Adjnoun	0.65	0.35	0.9	0.92	0.92
Facebook	0.68	0.32	0.8	0.79	0.81
Hamster	0.78	0.22	0.9	0.9	0.91
Jazz	0.82	0.18	0.92	0.9	0.93
Faa	0.7	0.3	0.82	0.85	0.88
Figeys	0.8	0.2	0.79	0.66	0.79
Lesmis	0.79	0.21	0.9	0.9	0.92
Moreno	0.69	0.31	0.85	0.87	0.87
Netscience	0.63	0.37	0.84	0.84	0.86
PowerGrid	0.7	0.3	0.52	0.59	0.59
Polbooks	0.74	0.26	0.85	0.88	0.9
USAir	0.84	0.16	0.89	0.88	0.89

**Table 4:** The Kendall correlation coefficients of the RSGNN model and three GNN models in 12 real networks.

Network	GCN	GAT	GraphSAGE	RSGNN
Adjnoun	0.84	0.5	0.77	0.92
Facebook	0.69	0.74	0.71	0.81
Hamster	0.84	0.7	0.73	0.91
Jazz	0.88	0.72	0.82	0.93
Faa	0.69	0.7	0.78	0.88
Figeys	0.73	0.66	0.64	0.79
Lesmis	0.71	0.65	0.73	0.92
Moreno	0.78	0.34	0.8	0.87
Netscience	0.65	0.73	0.77	0.86
PowerGrid	0.49	0.45	0.52	0.59
Polbooks	0.82	0.56	0.8	0.9
USAir	0.85	0.73	0.71	0.89

## Data Availability

Not applicable.
